# Comparison of Piperacillin and Tazobactam Pharmacokinetics in Critically Ill Patients with Trauma or with Burn

**DOI:** 10.3390/antibiotics11050618

**Published:** 2022-05-04

**Authors:** Daniel J. Selig, Kevin S. Akers, Kevin K. Chung, Adrian T. Kress, Jeffrey R. Livezey, Elaine D. Por, Kaitlin A. Pruskowski, Jesse P. DeLuca

**Affiliations:** 1 Walter Reed Army Institute of Research, Experimental Therapeutics, Silver Spring, MD 20910, USA; adrian.t.kress.mil@mail.mil (A.T.K.); elaine.d.por.mil@mail.mil (E.D.P.); jesse.p.deluca.mil@mail.mil (J.P.D.); 2 United States Army Institute of Surgical Research, San Antonio, TX 78234, USA; kevin.s.akers.mil@mail.mil (K.S.A.); kaitlin.a.pruskowski.civ@mail.mil (K.A.P.); 3 Department of Medicine, School of Medicine, Uniformed Services University of the Health Sciences, Bethesda, MD 20814, USA; kevin.chung@usuhs.edu (K.K.C.); jeffrey.livezey@usuhs.edu (J.R.L.)

**Keywords:** burns, critical illness, pharmacokinetics, piperacillin, tazobactam

## Abstract

Critical illness caused by burn and sepsis is associated with pathophysiologic changes that may result in the alteration of pharmacokinetics (PK) of antibiotics. However, it is unclear if one mechanism of critical illness alters PK more significantly than another. We developed a population PK model for piperacillin and tazobactam (pip-tazo) using data from 19 critically ill patients (14 non-burn trauma and 5 burn) treated in the Military Health System. A two-compartment model best described pip-tazo data. There were no significant differences found in the volume of distribution or clearance of pip-tazo in burn and non-burn patients. Although exploratory in nature, our data suggest that after accounting for creatinine clearance (CrCl), doses would not need to be increased for burn patients compared to trauma patients on consideration of PK alone. However, there is a high reported incidence of augmented renal clearance (ARC) in burn patients and pharmacodynamic (PD) considerations may lead clinicians to choose higher doses. For critically ill patients with normal kidney function, continuous infusions of 13.5–18 g pip-tazo per day are preferable. If ARC is suspected or the most stringent PD targets are desired, then continuous infusions of 31.5 g pip-tazo or higher may be required. This approach may be reasonable provided that therapeutic drug monitoring is enacted to ensure pip-tazo levels are not supra-therapeutic.

## 1. Introduction

Critical illness is associated with pathophysiologic changes that may result in the alteration of pharmacokinetics of antibiotics [1]. In particular, the increased volume of distribution (*Vd*) and augmented renal clearance (ARC) may necessitate higher antibiotic loading and maintenance doses to avoid sub-therapeutic concentrations [2]. A multitude of causes of critical illness may lead to systemic inflammation, increased capillary permeability and a subsequent leak of fluid and proteins to the extravascular space. Although mechanisms may differ for capillary leak based on the specific cause of critical illness, the resultant phenotypic changes are often similar [3,4]. Severe burn injury is a commonly cited cause of increased *Vd* and often higher antibiotic doses are recommended in this patient population [5]. However, sepsis alone may also cause increases in antibiotic *Vd*, and it is unclear if one specific cause of critical illness leading to similar phenotypic changes will have a larger impact on *Vd* over another.

Similarly, severe burn injury is a commonly cited cause of ARC, possibly related to increased cardiac output and blood flow to the kidneys [5]. Sepsis may also cause such physiologic changes and is also associated with ARC [6]. Furthermore, for antibiotics that are predominantly cleared by the kidneys, creatinine clearance (*CrCl*) is likely the largest determinant of achieving therapeutic concentrations and this remains independent of the mechanism of critical illness. This is strongly suggested by a multitude of population pharmacokinetic studies for piperacillin-tazobactam (pip-tazo), where *CrCl* is consistently a statistically significant covariate and predictor of achieving pharmacodynamic (PD) targets [7,8,9,10,11].

However, comparative data are often lacking; therefore, the additional effect of mechanism of critical illness after accounting for *CrCl* is difficult to quantify. For pip-tazo, Olbrisch et al. provides one of the only comparative datasets of piperacillin PK in burn compared to non-burn critically ill patients [12]. In this analysis, after accounting for differences in *CrCl*, piperacillin *CL* estimates were approximately 30% higher in the burn group (17.1 L/hr) compared to the non-burn group (13.22 L/hr). However, simulated probability of target attainment (PTA) was similar in both groups for achieving a free concentration above minimum inhibitory concentration for 100% of the dosing interval (100% fT > MIC). Of note, piperacillin *Vd* was not significantly different in burn and non-burn groups. 

Other than Olbrisch et al., there is a lack of supporting data to quantify the effect of mechanism of critical illness, particularly burn injury, on PK parameters and dose requirements. Furthermore, data presented by Olbrisch et al. are mixed, suggesting that burn increases piperacillin *CL* after accounting for *CrCl*; however, dose increases specific to burn may not be required. Therefore, we developed population PK models for pip-tazo using data from 19 critically ill patients (14 non-burn trauma and 5 burn). The aims of the study were to determine if there were significant differences in *Vd* or *CL* in burn versus non-burn trauma critically ill patients and whether increased doses of pip-tazo would be required in burn patients after accounting for *CrCl*.

## 2. Results

### 2.1. Patient Demographics

Patient demographics by burn status are summarized in Table 1. There were significant differences in age, weight and *CrCl* between the groups with the burn population being generally younger, heavier and possessing higher *CrCl*. *CrCl* estimates were high in both groups but were plausible given the known prevalence of ARC in critically ill burn and non-burn trauma populations [13,14,15]. Reported concurrent injuries are summarized in Table S1 (Supplementary Materials).

### 2.2. Population Pharmacokinetic Models

- Base Model

The model building processes for piperacillin and tazobactam are summarized in Tables S2 and S3 (Supplementary Material), respectively. The data for both piperacillin and tazobactam were best described by two-compartment models with proportional error model. The two-compartment model was parameterized with terminal clearance  CL, intra-compartmental clearance  Q, volume of the central compartment $V_{c}$ and volume of the peripheral compartment $V_{p}$. Between subject variability (BSV) was not estimated for Q or $V_{p}$.

- Covariate Model

Parameter estimates of the final model are summarized in Table 2 and Table 3. Covariate exploratory plots with random effects generated from the base model demonstrated trends between ηCL and *CrCl.* Creatinine clearance was found to be a significant covariate for both piperacillin and tazobactam *CL* (*p* = 0.009 and *p* = 0.001, respectively) explaining 8.71% and 23.68% of the variability on *CL* for pip-tazo, respectively. For tazobactam, the power exponent was estimated to be 0.77, which was consistent with that of Chandorkar et al. (0.67). Given that Chandorkar et al.’s dataset included significantly more patients with a wider range of *CrCl’s*, we fixed the *CrCl* estimate for tazobactam to 0.67. Urine output was not found to be a significant covariate for pip-tazo *CL*, but it trended toward significance on piperacillin *CL*. As *CrCl* better explained pip-tazo *CL* and urine output may be confounded by many factors in critically ill patients [16], *CrCl* was chosen over urine output in the covariate model. Notably, covariate plots with burn as a category or TBSA showed no trends with ηCL or $\eta V_{c}$ (Figures S1 and S2, Supplemental Material). Correspondingly, for both piperacillin and tazobactam, neither burn as a categorical covariate nor TBSA were significant covariates for *CL*, $V_{c}$ or $V_{p}$. Albumin trended toward significance as a covariate on $V_{p}$ for both piperacillin and tazobactam (*p* = 0.078 and *p* = 0.089, respectively). The final equation for CL for piperacillin was as follows.

(1) $$\begin{array}{c} CL_{i}=17.56\cdot {\left(\frac{CrCl}{130}\right)}^{0.65}\cdot e^{\eta CL_{i}} \end{array}$$

Moreover, for tazobactam, it was the following.

(2) $$\begin{array}{c} CL_{i}=11.54\cdot {\left(\frac{CrCl}{130}\right)}^{0.67}\cdot e^{\eta CL_{i}} \end{array}$$

- Validation of Final Model

**Figure 1:**
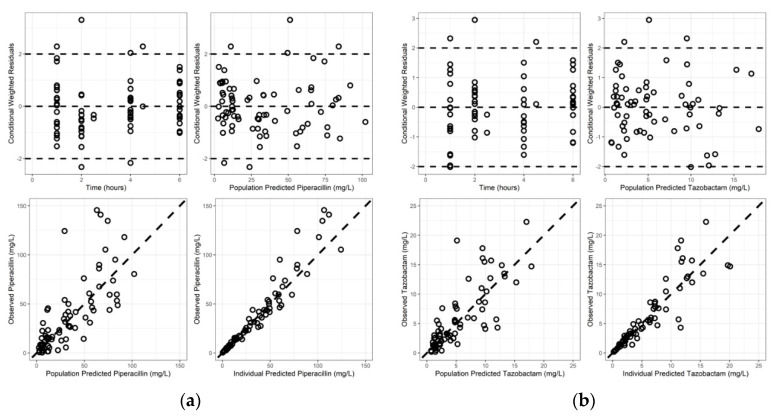
Goodness of fit plots for final models: (**a**) piperacillin; (**b**) tazobactam.

Goodness-of-fit plots revealed model predictions to be randomly scattered around the line of unity. There were no significant trends in plots of conditional weighted residuals versus time or conditional weighted residuals versus predicted concentrations (Figure 1a,b). Individual fit plots demonstrated both the population and individual predicted concentrations fit reasonably well to the observed data (Figure S3, Supplementary Material). The histograms of conditional weighted residuals and BSV random effects were consistent with normally distributed data centered at 0 (Figure S4, Supplementary Material). Visual predictive checks demonstrated that the observed data and quantiles fell within the simulated 95% CI’s (Figure S5, Supplementary Material). Plots of random effects vs. covariates appropriately demonstrate eliminated or diminished trends upon inclusion of the covariate in the final model (Figure S5, Supplementary Material). 

### 2.3. Probability of Target Attainment

**Figure 2:**
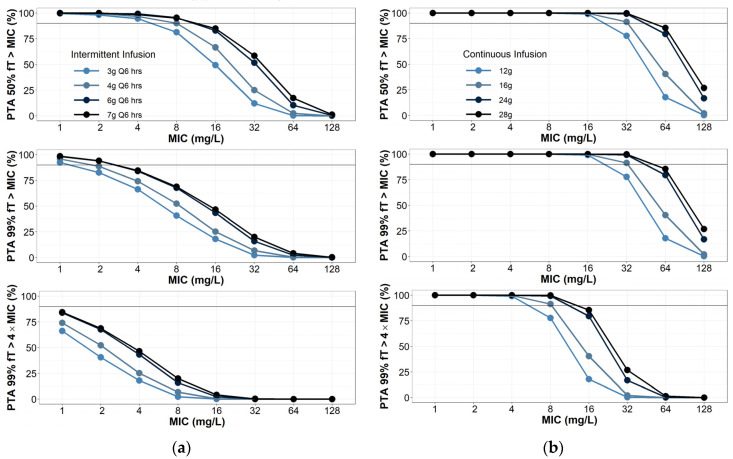
Probability of target attainment for (**a**) piperacillin intermittent infusions; (**b**) continuous infusions. Intermittent infusions assumed to be infused over 30 min. Creatinine clearance was randomly selected between 100 and 130 mL/min, with 1000 virtual patients simulated per dosing group.

The probability of target attainment is summarized in Figure 2 and Table S4 (Supplementary Material). The results of intermittent infusion simulations were consistent with those previously reported across multiple patient populations (general critical care [10], burn [11] and hospitalized [17]). Generally, intermittent infusions of 4 g piperacillin every 6–8 h would not be adequate to target pathogens with an MIC of 8–16 mg/L even for the most lenient PD target (50% fT > MIC). Continuous infusions PTA simulations were also consistent with previous reports [7,8,18]. Lenient PD targets (50–100% fT > MIC) are consistently met with daily doses as low as 12 g. However, when considering 100% fT > 4 × MIC as the ideal PD target, no dose simulated achieved 90% for PTA success. Doses of 28 g per day approached the PTA threshold for success. Ranging the assumed fraction unbound from 0.7 to 0.5 did not have a clinically significant effect on achieving PTA (Table S4, Supplementary Material).

## 3. Discussion

We have developed population PK models for piperacillin and tazobactam in critically ill patients with or without burn injury. We found that neither burn nor TBSA was a significant covariate on *CL*, *V_c_* or *V_p_*. Although our work is exploratory in nature and limited by a convenience sample, these results in combination with the literature review suggest that burn injury, even with high TBSA, should not drive dosing decisions based on PK considerations alone. 

Although there appears to be a general literature consensus that antibiotics, including pip-tazo, should be prescribed at higher doses in burn patients, this conclusion is often made based off of data lacking a control group [19,20]. For example, Jeon et al. found that short intermittent doses of 4.5 g pip-tazo every 8 h in critically ill burn patients would not be adequate to achieve a lenient PD target of 50% ft > MIC [11]. However, this same finding was noted by Merino-Bohórquez et al. [17] in a cohort of non-critically ill patients and Alobaid et al. in a more general critically ill population [10]. In fact, when normalizing *CrCl* to 130 mL/min, as in our study, the piperacillin *CL* estimates were comparable to all of these studies despite significant differences in patient populations (16.35 L/hr [11], 15.11 L/hr [17], 18.2 L/hr [10] and 17.56 L/hr—current study). Of note, not all of these studies reported confidence intervals; however, Jeon et al. and the current study found confidence intervals of 13.4–18.4 L/hr and 14.38–20.73 L/hr, respectively. This suggests there is unlikely to be a statistically significant difference in piperacillin *CL* amongst these populations after accounting for estimated *CrCl*. 

Although the population estimates for piperacillin *CL* are similar in the cohorts reported above, population estimates from critically ill or hospitalized patients may generally be prone to bias, and there are non-burn cohorts that do have lower estimates of piperacillin *CL* [21]. For example, Hamada et al. reports a piperacillin CL of 11.5 L/hr normalized to a *CrCl* of 130 mL/min in a cohort of patients with community-acquired pneumonia [22]. Furthermore, Olbrisch et al. provide one of the only piperacillin PK analyses internally comparing critically ill patients with and without burn. Olbrisch et al. presents mixed evidence on whether prescribed doses of piperacillin should be higher in burn patients even after accounting for *CrCl*. After normalizing *CrCl* between the two study groups (to 113 mL/min per study report), piperacillin *CL* estimates were approximately 30% higher in the burn group (17.1 L/hr) compared to the non-burn group (13.22 L/hr). There is physiologic plausibility to this, as piperacillin may have a saturable elimination pathway and burn is associated with upregulated metabolism [19,23]. Nevertheless, even with the difference in *CL* observed between burn and non-burn patients in Olbrisch et al., PTA was similar for both groups when considering 100% fT > MIC. When considering 100% fT > 4 × MIC, PTA was significantly improved in the non-burn population. However, optimal PD targets for piperacillin are currently in question [24,25] and a 30% difference in clearance between two critically ill populations is not entirely unexpected given the potential for biased estimates, reliance on C-G for *CrCl* estimates, male predominance in the burn group (74% male burn and 50% male non-burn) and significant difference in age (mean 72.7 years non-burn and 48.5 years burn). Burn, younger age and male sex are risks factors for ARC [13,14]. Therefore, with a more accurate characterization of kidney function and ARC, it is possible that the observed difference in *CL* may not be as significant after accounting for *CrCl*.

Regarding *Vd*, piperacillin *Vd* is likely to be higher in both burn and non-burn critically ill patients compared to healthy volunteers. Whereas healthy volunteers are reported to have steady state *Vd* in the range of 6–12 L [23,26,27], many authors have reported Vd in critically ill patients to be in the range of 25–50 L [8,10,11,12,18]. However, it is unclear if critically ill patients with burn have a higher Vd compared to other critically ill populations. The estimates of *Vd* in burn patients are reported to be 19.5 L, 28.1 L and 41.4 L by Bourget et al. [28], Olbrisch et al. and Jeon et al., respectively. The only one of these studies that had an internal comparator group of non-burn critically ill patients was Olbrisch et al., reporting a *Vd* of 22.9 L in non-burn patients, notably not statistically significantly different from burn patients within the study. Furthermore, several studies report Vd in non-burn critically ill patients similar to those from Bourget et al., Olbrisch et al. and Jeon et al. [7,8,10]. Tsai et al. and Felton et al. note significantly smaller *Vd* in non-burn critically ill patients (14.5 L and 7.54 L), suggesting there could be a larger piperacillin *Vd* imparted by burn injury. 

Nevertheless, even if *Vd* were to be significantly elevated in burn patients compared to other critically ill populations, there would likely be no required changes in piperacillin dosing as a result. We previously performed simulations assuming large changes in *Vd* for both imipenem and meropenem (both %fT > MIC antibiotics) [29,30]. We found that significantly elevated *Vd* was associated with improved PTA. This finding is grounded soundly in pharmacokinetic principles. The equation for half-life [31] is as follows.

(3) $$\begin{array}{c} Half-Life=0.693\cdot \frac{Vd}{CL} \end{array}\;$$

Therefore, if *CL* is held constant, half-life will increase with an increase in *Vd*, leading to a shallower PK curve, with higher concentrations through the end of the dosing interval (but also with a smaller maximum concentration). Physiologically, this is also plausible, as a significant disruption in capillary permeability may lead to antibiotics accumulating in the extravascular spaces, which are then slowly resorbed. As steady state for piperacillin, imipenem and meropenem may be achieved in one or two doses, and the increased *Vd* at a given *CL* will lead to higher concentrations towards the end of the curve. For beta-lactams and carbapenems, an increased *Vd* likely imparts a greater chance of PTA success. This is contrary to aminoglycosides, where efficacy is more correlated to maximum concentrations, and large increases in *Vd* may require higher loading doses [32].

The limitations to our study include reliance on a convenience sample. As such, our results should be considered exploratory and confirmed with larger, controlled studies. Furthermore, our non-burn control group consisted of critically ill trauma patients; therefore, the results may not be broadly applicable across all critically ill populations. The use of two-compartment models in our study may be limited by inadequate sampling in the distribution phases; however, piperacillin and tazobactam have both been adequately described by two-compartment models and our parameter estimates are plausible and within the range of those reported in the literature. The assumption of piperacillin fraction unbound of 0.7 was a practical simplification. Nevertheless, this was consistent with the fraction unbound observed in our study (0.64 overall, 0.6 non-burn trauma and 0.75 burn). In addition, PTA analyses assuming fraction unbounds of 0.5, 0.6 or 0.7 led to similar results and would not change dosing recommendations. Finally, there are many factors that may theoretically affect pip-tazo PK in critically ill populations such as concomitant medications and the overall score and individual components of critical illness calculators. Our study did not discriminate enrollment or attempt to stratify on such factors, as such, our model does not account for their effects. This may introduce additional confounding bias beyond the use of convenience sample in our burn vs. non-burn PK comparison. However, to our knowledge, there is scant literature providing quantitative estimates of these factors on pip-tazo *CL*, and they are not usually found to be significant covariates in pip-tazo population PK models [7,8,9,10,11,33,34]. The most consistent statistically significant covariate for pip-tazo CL is *CrCl*, and this covariate is typically the main determinant of pip-tazo dosing. Our model had similar estimates for the effect of *CrCl* as reported in the literature. Therefore, although our model does not explain all confounding factors, it is reasonable and does provide significant insight into the comparative PK of burn and non-burn trauma patients. 

## 4. Materials and Methods

### 4.1. Data 

**Figure 3:**
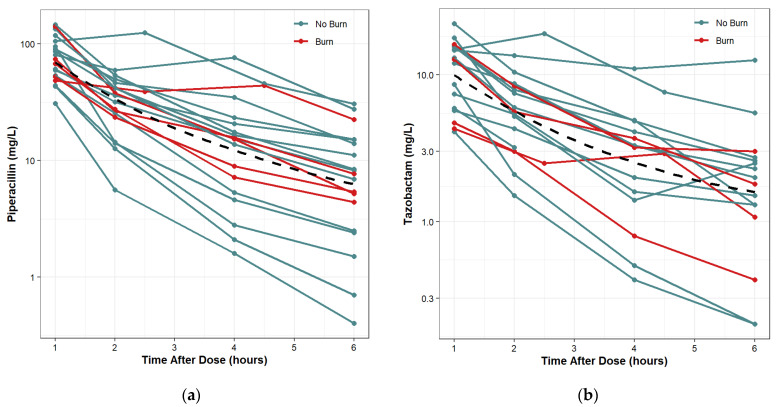
Time-concentration plots with LOESS trend lines: (**a**) piperacillin; (**b**) tazobactam (right).

Protocol and associated documents that include informed consent forms were reviewed and approved by IRB at the United States Army Medical Research and Development Command (MRDC; Fort Detrick, MD). De-identified patient data were obtained from an IRB-approved protocol at the USAISR Burn Center and Brooke Army Medical Center (BAMC) Surgical Trauma Intensive Care Unit (STICU). There were a total of 19 patients, 14 with no burn injury and 11 with burn injury. The most commonly prescribed dose was 4.5 g pip-tazo every 6 h (N = 11 patients). Eight patients received 3.375 g pip-tazo every 6 h (6 non-burn and 2 burn). All doses were infused over 0.5 h except for one patient who had an infusion time of 0.62 h. For each patient, free and total plasma pip-tazo samples were collected at steady state from 1 to 6 h post initiation of the infusion. There was one set of samples for each patient drawn prior to the dose; however, the timing in relation to the previous dose was unknown, so these samples were not used for the population PK analysis. For piperacilln, all 19 subjects were included, and there was a total of 76 post-dose concentration observations. For tazobactam, 2 subjects did not have adequate concentration sampling and were excluded from the analysis. One patient included in the tazobactam analysis had 2 concentration observations less than the lower limit of quantification and these concentrations were assumed to be missing. A total of 17 patients and 66 post-dose concentration observations were used for the tazobactam model. For piperacillin, all 19 patients had 4 post-dose plasma observations. For tazobactam, 16 patients had 4 post-dose plasma observations and 1 patient had 2 post-dose plasma observations. The time-concentration data included in the analysis are summarized in Figure 3a,b. There were no missing demographic data.

### 4.2. High-Performance Liquid Chromatography (HPLC)

Piperacillin and tazobactam concentrations from patient samples were determined by HPLC using a method previously validated in our laboratory. A Dionex 3000 HPLC system (Dionex, Thermo-Fisher Inc., Sunnyvale, CA, USA) with UV detection at 190 nm and 210 nm was used for analysis. Briefly, mobile phase A consisted of 5mM phosphate buffer at pH 3.0, and mobile phase B was 100% MeCN. These were run at a flow rate of 1 mL/min, applying different gradients of mobile phase B. For piperacillin, mobile phase B was increased from 30% to 45% over 14 min. For tazobactam, mobile phase B increased from 5% to 30% over 10 min. The stationary phase was a 150 mm octadecyl column (Luna 5u C18 100A 150 × 4.6mm; Phenomenex, Torrance, CA, USA). This resulted in retention times for piperacillin and tazobactam of approximately 5.5 min and approximately 10.5 min for oxacillin (internal standard, IS). Standard curves were constructed for each analyte by injecting reference solutions of known concentrations of analyte and IS. Peak areas of the eluted drugs were integrated, and concentrations were quantified using peak area ratios of analyte to IS. Linearity was confirmed from 0.50 µg/mL to 25.0 µg/mL, with the mean (±SD) between-day calibration curve regression r^2^ = 0.9907 ± 1.729 for piperacillin and 0.9794 ± 1.215 for tazobactam. Between-day coefficients of variation at 0.5 µg/mL and 25.0 µg/mL were 16% and 11%, respectively, for piperacillin and 19% and 11% for tazobactam. The limit of detection for the assay was 0.010 µg. Solid Phase Extraction (SPE) columns (C18 Sep-Pak, Waters Corporation, Milford, MA, USA.) were conditioned with a 1:1 mixture solution of 100% MeOH in mobile phase A. Samples were prepared by adding 3 µg of IS to 300 µL of plasma, from which 200 µL was loaded onto the SPE columns. The SPE columns containing sample were washed twice with 1mL of mobile phase A. Analytes were eluted using 800 µL 100% MeOH and evaporated to dryness under N2. After reconstitution in 200 µL of mobile phase A, 50μL of aliquots was injected into the HPLC for analysis. The concentration of drug in each sample was determined by regression analysis of the peak area ratios.

### 4.3. Population Pharmacokinetic Modelling and Simulations

Population pharmacokinetic modelling and simulations were performed in Pumas (version 1.1) [35]. The first order conditional estimation method with interaction (FOCEI) was used to estimate population parameters. Data preparation, exploratory analysis and graphs were performed in either Pumas or R (version 3.6.1). Data from all patients, both those with or without burn, were modelled simultaneously.

- Base Model

One and two-compartment models were explored for this study. Between subject variability (BSV) was modeled using an exponential error model under the assumption that pharmacokinetic parameters are distributed log normally. Parameters generally took the following form:

(4) $$\begin{array}{c} \theta_{i}=tv\theta \cdot e^{\eta_{i}} \end{array}\;$$

where $\theta_{i}$ is the post hoc estimated parameter value for individual i*,* tvθ is the population mean parameter and $\eta_{i}\;\sim \;\left(0,\;\omega^{2}\right)\;$ is the between subject random effects for individual i. The selection of the base model was based on the likelihood ratio test (LRT) with  α=0.05, plausibility and precision of parameter estimates and diagnostic plots.

- Covariate Model

Covariates evaluated were total body weight (WT), lean body mass (LBM), creatinine clearance (CrCl), 24 h urine output (UOP), age, total burn surface area (TBSA), burn as a categorical covariate and serum albumin (ALBUM). Creatinine Clearance was estimated by the Cockcroft-Gault (C-G) equation [36] and LBM was calculated using Janmahasatian’s formula [37]. Continuous covariates except for CrCl, which was scaled to 130 mL/min, were modeled as follows:

(5) $$\begin{array}{c} \theta_{i}=tv\theta \cdot {\left(\frac{COV}{COV_{median}}\right)}^{\theta_{COV}}\;\; \end{array}\;$$

or

(6) $$\begin{array}{c} \theta_{i}=tv\theta +\theta_{COV}\cdot COV \end{array}$$

where is $\theta_{i}$ is the PK parameter in individual i, tvθ is the typical value of the PK parameter at the median value of the covariate ($COV_{median}$), COV is the covariate observed in individual i and $\theta_{COV}$ is the power or slope estimate for the covariate. 

Categorical covariates were modeled as follows

(7) $$\begin{array}{c} \theta_{i}=tv\theta \cdot \left(1+\theta_{COV}\cdot COV\right)\; \end{array}$$

where  COV is binary (coded as 0 or 1), tvθ represents the typical value of the PK parameter when COV=0 and $\theta_{COV}$ represents the proportional change in tvθ when COV=1. Covariate modelling was performed with a forward addition process. A decrease of at least 3.84 units (α=0.05, df=1) in the objective function value (OFV) was considered statistically significant.

- Final Model Qualification

Final model qualification included the examination of standard goodness-of-fit plots, precision of parameter estimates based on inference and bootstrap methods (N = 500 runs) and visual predictive checks (200 replicates). External model evaluation included comparing typical values of PK parameter estimates of our final model to those in the existing literature.

- Monte Carlo Simulations

Monte Carlo simulations were performed only for piperacillin. Clinical tazobactam targets are not as well established and Kalaria et al. provided a comprehensive discussion on tazobactam PK and dosing in critically ill patients [9]. The probability of target attainment (PTA) was considered a surrogate for efficacy and was explored using 3 commonly cited PD targets (50% fT > MIC, 100% fT > MIC and 100% fT > 4 × MIC). Creatinine clearance was randomly selected from 100 to 130 mL/min. For each scenario, 1000 patient concentration-time profiles were simulated without residual unexplained variability (RUV). The percentage of simulated patients that achieved the various PD targets was calculated at MIC’s ranging from 1 mg/L to 128 mg/L with PTA > 90% considered acceptable. The pip-tazo breakpoint for pseudomonas (16 mg/L) as determined by the European Committee on Antimicrobial Susceptibility Testing (EUCAST) [38] and the Clinical and Laboratory Standards Institute (CLSI) [39] was considered the ideal MIC target. Free concentrations were assumed to be 0.7 times that of the simulated total concentrations [40]. PTA was also calculated at the breakpoint of 16 mg/L assuming fraction unbounds of 0.6 and 0.5.

## 5. Conclusions

Although exploratory in nature, our data suggest that after accounting for estimated (*CrCl*), doses of pip-tazo would not need to be increased for burn patients on the consideration of PK alone. However, there may be a higher incidence of augmented renal clearance (ARC) in burn patients compared to other critically ill populations and pharmacodynamic (PD) considerations may lead clinicians to choose higher dosing regimens of pip-tazo in burn patients. For critically ill patients with normal kidney function, we recommend continuous infusions of 13.5–18 g pip-tazo per day. If ARC is suspected or the most stringent PD targets are desired, then continuous infusions of 31.5 g pip-tazo or higher may be required. This approach may be reasonable provided therapeutic drug monitoring is enacted to ensure pip-tazo levels are not supratherapeutic.

## Figures and Tables

**Table 1:** Demographics of the total, burn and no-burn populations.

Category	All (N = 19) ^1^	No Burn Trauma (N = 14)	Burn (N = 5)
Age (years)	38.3 ± 16.34	42.86 ± 16.7	25.6 ± 4.62
Sex	16 male, 3 female	11 male, 3 female	5 male, 0 female
Weight (kg)	88 ± 26.43	82.44 ± 20.66	103.56 ± 36.68
Lean Body Mass (kg)	61.03 ± 13.12	58.27 ± 12.05	68.76 ± 14.18
Serum Creatinine (mg/dL)	0.74 ± 0.25	0.73 ± 0.29	0.77 ± 0.15
Creatinine Clearance (mL/min)	176.56 ± 63.58	164.46 ± 63.46	210.41 ± 56.2
Urine Output (mL)	2802.4 ± 1443.5	3035.7 ± 1542.7	2149.2 ± 846.1
Blood Urea Nitrogen	19.07 ± 8.48	18 ± 7.6	22.04 ± 10.1
Albumin (g/dL)	2.43 ± 0.63	2.58 ± 0.6	2.02 ± 0.58
Total Burn Surface Area (%)	–	–	38.2 ± 28.53
Piperacillin Fraction Unbound	0.64 ± 0.33	0.6 ± 0.33	0.75 ± 0.3
Tazobactam Fraction Unbound	0.51 ± 0.4	0.48 ± 0.3	0.6 ± 0.61

^1.^ No patients were on vasopressors on the day of sampling.

**Table 2:** Pharmacokinetic parameters for final piperacillin model.

Parameter	FOCEI Estimate (%RSE)	FOCEI 95% CI	Bootstrap Estimate (95% CI)
*CL* (L/hr)	17.56 (9.24)	14.38–20.73	17.56 (13.66–21.61)
*V_c_* (L)	33.59 (16.21)	22.92–44.27	33.59 (23.4–45.02)
*Q* (L/hr)	6.8 (29.97)	2.81–10.8	6.8 (3.1–11.15)
*V_p_* (L)	10.5 (20.44)	6.29–14.7	10.5 (7.91–23.97)
Covariates on CL			
*CrCl* (power)	0.65 (20.63)	0.38–0.91	0.65 (0.27–1.11)
Random Effects			
ω^2^ *CL*	0.17 (27.35)	0.08–0.27	0.17 (0.068–0.26)
ω^2^ *V_c_*	0.34 (44.14)	0.046–0.63	0.34 (0.013–0.65)
η-shrinkage *CL*: 1.39%, η-shrinkage V_c_: 14.9% Pearson’s correlation coefficients: *η-V_c_* & *η-CL*: 0.065			
Residual Unexplained Variability			
Proportional Error	0.24 (10)	0.19–0.28	0.24 (0.18–0.28)
ϵ-shrinkage: 20.27% 19 subjects, 76 total concentration observations, OFV = 552.3, condition number = 83569.8			

**Table 3:** Pharmacokinetic parameters for final tazobactam model.

Parameter	FOCEI Estimate (%RSE)	FOCEI 95% CI	Bootstrap Estimate (95% CI)
*CL* (L/hr)	11.54 (12.75)	8.65–14.42	11.54 (5.22–14.28)
*V_c_* (L)	20.12 (36.69)	5.65–34.49	20.21 (1.51–31.44)
*Q* (L/hr)	9.84 (51.93)	0–19.86	9.84 (3.72–15.74)
*V_p_* (L)	15.93 (14.64)	11.36–20.49	15.93 (7.6–23.52)
Covariates on CL			
CrCL (power)—fixed	0.67	–	0.67
Random Effects			
ω^2^ *CL*	0.13 (44.03)	0.02–0.23	0.13 (0.037–0.24)
η-shrinkage CL: 0.28%			
Residual Unexplained Variability			
Proportional Error	0.29 (12.18)	0.21–0.35	0.29 (0.21–0.34)
ϵ-shrinkage: 10.86% 17 subjects, 66 total concentration observations, OFV = 255.22, condition number = 83,243			

## Data Availability

Data presented in this article cannot be shared. For any other questions, please contact the corresponding author.

## Supplementary Materials

The following supporting information can be downloaded at: https://www.mdpi.com/article/10.3390/antibiotics11050618/s1, Table S1: Concurrent Reported Injuries; Table S2: Piperacillin Model Building; Table S3: Tazobactam Model Building; Table S4: Sensitivity Analysis of Fraction Unbound on Probability of Target Attainment; Figure S1: Clearance Covariate Exploratory Plots; Figure S2: Volume Covariate Exploratory Plots; Figure S3: Individual Goodness of Fit Plots; Figure S4: Summary of Residual and Between Subject Variability; Figure S5: Visual Predictive Checks

## References

[1] Blot S.I., Pea F., Lipman J. (2014). The effect of pathophysiology on pharmacokinetics in the critically ill patient—Concepts appraised by the example of antimicrobial agents. Adv. Drug Deliv. Rev..

[2] Varghese J.M., Roberts J.A., Lipman J. (2011). Antimicrobial pharmacokinetic and pharmacodynamic issues in the critically ill with severe sepsis and septic shock. Crit. Care Clin..

[3] Bichon A., Bourenne J., Gainnier M., Carvelli J. (2021). Capillary leak syndrome: State of the art in 2021. Rev. de Médecine Interne.

[4] Wollborn J., Hassenzahl L.O., Reker D., Staehle H.F., Omlor A.M., Baar W., Kaufmann K.B., Ulbrich F., Wunder C., Utzolino S. (2021). Diagnosing capillary leak in critically ill patients: Development of an innovative scoring instrument for non-invasive detection. Ann. Intensive Care.

[5] Pruskowski K.A. (2021). Pharmacokinetics and Pharmacodynamics of Antimicrobial Agents in Burn Patients. Surg. Infect..

[6] Luo Y., Wang Y., Ma Y., Wang P., Zhong J., Chu Y. (2021). Augmented Renal Clearance: What Have We Known and What Will We Do?. Front. Pharmacol..

[7] Klastrup V., Thorsted A., Storgaard M., Christensen S., Friberg L.E., Obrink-Hansen K. (2020). Population Pharmacokinetics of Piperacillin following Continuous Infusion in Critically Ill Patients and Impact of Renal Function on Target Attainment. Antimicrob. Agents Chemother..

[8] Dhaese S.A.M., Roberts J.A., Carlier M., Verstraete A.G., Stove V., De Waele J.J. (2018). Population pharmacokinetics of continuous infusion of piperacillin in critically ill patients. Int. J. Antimicrob. Agents.

[9] Kalaria S.N., Gopalakrishnan M., Heil E.L. (2020). A Population Pharmacokinetics and Pharmacodynamic Approach To Optimize Tazobactam Activity in Critically Ill Patients. Antimicrob. Agents Chemother..

[10] Alobaid A.S., Wallis S.C., Jarrett P., Starr T., Stuart J., Lassig-Smith M., Mejia J.L., Roberts M.S., Roger C., Udy A.A. (2017). Population Pharmacokinetics of Piperacillin in Nonobese, Obese, and Morbidly Obese Critically Ill Patients. Antimicrob. Agents Chemother..

[11] Jeon S., Han S., Lee J., Hong T., Paek J., Woo H., Yim D.S. (2014). Population pharmacokinetic analysis of piperacillin in burn patients. Antimicrob. Agents Chemother..

[12] Olbrisch K., Kisch T., Thern J., Kramme E., Rupp J., Graf T., Wicha S.G., Mailander P., Raasch W. (2019). After standard dosage of piperacillin plasma concentrations of drug are subtherapeutic in burn patients. Naunyn Schmiedebergs Arch. Pharmacol..

[13] Baptista J.P., Martins P.J., Marques M., Pimentel J.M. (2020). Prevalence and Risk Factors for Augmented Renal Clearance in a Population of Critically Ill Patients. J. Intensive Care Med..

[14] Loirat P., Rohan J., Baillet A., Beaufils F., David R., Chapman A. (1978). Increased glomerular filtration rate in patients with major burns and its effect on the pharmacokinetics of tobramycin. N. Engl. J. Med..

[15] Mulder M.B., Eidelson S.A., Sussman M.S., Schulman C.I., Lineen E.B., Iyenger R.S., Namias N., Proctor K.G. (2019). Risk Factors and Clinical Outcomes Associated With Augmented Renal Clearance in Trauma Patients. J. Surg. Res..

[16] Legrand M., Payen D. (2011). Understanding urine output in critically ill patients. Ann. Intensive Care.

[17] Merino-Bohorquez V., Docobo-Perez F., Valiente-Mendez A., Delgado-Valverde M., Camean M., Hope W.W., Pascual A., Rodriguez-Bano J. (2021). Population Pharmacokinetics of Piperacillin in Non-Critically Ill Patients with Bacteremia Caused by Enterobacteriaceae. Antibiotics.

[18] Dhaese S.A.M., Farkas A., Colin P., Lipman J., Stove V., Verstraete A.G., Roberts J.A., De Waele J.J. (2019). Population pharmacokinetics and evaluation of the predictive performance of pharmacokinetic models in critically ill patients receiving continuous infusion meropenem: A comparison of eight pharmacokinetic models. J. Antimicrob. Chemother..

[19] Udy A.A., Roberts J.A., Lipman J., Blot S. (2018). The effects of major burn related pathophysiological changes on the pharmacokinetics and pharmacodynamics of drug use: An appraisal utilizing antibiotics. Adv. Drug Deliv. Rev..

[20] Cota J.M., FakhriRavari A., Rowan M.P., Chung K.K., Murray C.K., Akers K.S. (2016). Intravenous Antibiotic and Antifungal Agent Pharmacokinetic-Pharmacodynamic Dosing in Adults with Severe Burn Injury. Clin. Ther..

[21] Cunio C.B., Uster D.W., Carland J.E., Buscher H., Liu Z., Brett J., Stefani M., Jones G.R.D., Day R.O., Wicha S.G. (2020). Towards precision dosing of vancomycin in critically ill patients: An evaluation of the predictive performance of pharmacometric models in ICU patients. Clin. Microbiol. Infect..

[22] Hamada Y., Takahashi S., Hirayama T., Sunakawa K., Kuroyama M. (2013). Population pharmacokinetics of tazobactam/piperacillin in Japanese patients with community-acquired pneumonia. Jpn. J. Antibiot..

[23] Landersdorfer C.B., Bulitta J.B., Kirkpatrick C.M., Kinzig M., Holzgrabe U., Drusano G.L., Stephan U., Sorgel F. (2012). Population pharmacokinetics of piperacillin at two dose levels: Influence of nonlinear pharmacokinetics on the pharmacodynamic profile. Antimicrob. Agents Chemother..

[24] Barreto E.F., Webb A.J., Pais G.M., Rule A.D., Jannetto P.J., Scheetz M.H. (2021). Setting the Beta-Lactam Therapeutic Range for Critically Ill Patients: Is There a Floor or Even a Ceiling?. Crit. Care Explor..

[25] Roger C., Louart B. (2021). Beta-Lactams Toxicity in the Intensive Care Unit: An Underestimated Collateral Damage?. Microorganisms.

[26] Felton T.W., Ogungbenro K., Boselli E., Hope W.W., Rodvold K.A. (2018). Comparison of piperacillin exposure in the lungs of critically ill patients and healthy volunteers. J. Antimicrob. Chemother..

[27] Bulitta J.B., Kinzig M., Jakob V., Holzgrabe U., Sorgel F., Holford N.H. (2010). Nonlinear pharmacokinetics of piperacillin in healthy volunteers--implications for optimal dosage regimens. Br. J. Clin. Pharmacol..

[28] Bourget P., Lesne-Hulin A., Le Reveille R., Le Bever H., Carsin H. (1996). Clinical pharmacokinetics of piperacillin-tazobactam combination in patients with major burns and signs of infection. Antimicrob. Agents Chemother..

[29] Por E.D., Akers K.S., Chung K.K., Livezey J.R., Selig D.J. (2021). Population Pharmacokinetic Modeling and Simulations of Imipenem in Burn Patients With and Without Continuous Venovenous Hemofiltration in the Military Health System. J. Clin. Pharmacol..

[30] Selig D.J., Akers K.S., Chung K.K., Pruskowski K.A., Livezey J.R., Por E.D. (2021). Meropenem pharmacokinetics in critically ill patients with or without burn treated with or without continuous veno-venous haemofiltration. Br. J. Clin. Pharmacol..

[31] Hallare J., Gerriets V. (2022). Half Life.

[32] Lee C., Walker S.A.N., Walker S.E., Seto W., Simor A., Jeschke M. (2017). A prospective study evaluating tobramycin pharmacokinetics and optimal once daily dosing in burn patients. Burns.

[33] Sime F.B., Lassig-Smith M., Starr T., Stuart J., Pandey S., Parker S.L., Wallis S.C., Lipman J., Roberts J.A. (2019). Population Pharmacokinetics of Unbound Ceftolozane and Tazobactam in Critically Ill Patients without Renal Dysfunction. Antimicrob. Agents Chemother..

[34] Tsai D., Stewart P., Goud R., Gourley S., Hewagama S., Krishnaswamy S., Wallis S.C., Lipman J., Roberts J.A. (2016). Pharmacokinetics of Piperacillin in Critically Ill Australian Indigenous Patients with Severe Sepsis. Antimicrob. Agents Chemother..

[35] Rackauckas C., Ma Y., Noack A., Dixit V., Mogensen P.K., Bryne S., Maddhashiya S., Santiago Calderon J.B., Nyberg J., Gobburu J.V.S. (2020). Accelerated Predictive Healthcare Analytics with Pumas, a High Performance Pharmaceutical Modeling and Simulation Platform. bioRxiv.

[36] FDA (2020). Guidance for Industry Pharmacokinetics in Patients with Impaired Renal Function—Study Design, Data Analysis, and Impact on Dosing. https://www.fda.gov/media/78573/download.

[37] Janmahasatian S., Duffull S.B., Ash S., Ward L.C., Byrne N.M., Green B. (2005). Quantification of lean bodyweight. Clin. Pharmacokinet..

[38] Testing TECoAS (2021). Breakpoint Tables for Interpretation of MICs and Zone Diameters. Version 11.0. http://www.eucast.org.

[39] Institute CaLS (2021). CLSI Supplement M100. http://em100.edaptivedocs.net/GetDoc.aspx?doc=CLSI%20M100%20ED31:2021&scope=user.

[40] Pfizer (1993). Piperacillin and Tazobactam Package Insert. https://www.accessdata.fda.gov/drugsatfda_docs/label/2017/050684s88s89s90_050750s37s38s39lbl.pdf.

