# Supplementary material for: Comparison of Piperacillin and Tazobactam Pharmacokinetics in Critically Ill Patients with Trauma or with Burn
Source: Antibiotics (Basel). 2022 May 4;11(5):618. doi: 10.3390/antibiotics11050618 (PMC9138153; doi:10.3390/antibiotics11050618)

Supplemental Material

Table S1: Concurrent Reported Injuries

Patient	Injuries
12EMNA	Motor vehicle accident with subarachnoid hemorrhage, pneumocephalus, multiple facial and skull fractures
67ED2Z	Motor vehicle accident with head on collision
6FQ9Y0	Motor vehicle collision with pedestrian
6K62IV	Traumatic complete right lower lobe collapse, right scapular fracture, displaced ribs and rib fractures, multifocal intraparenchymal hemorrhages
6L8TCE	Motorcycle collision, required chest wall needle decompression, C6 spinal fracture, right scapular fracture, right clavicle fracture, right chest flail segment and grade 2 liver injury
8I1155	Motor vehicle collision, 8% TBSA with left acetabular, femoral, ankle, digit transverse process fractures. Sustained partial and full thickness burns to left hand, left forearm, partial left upper arm
9SOZIE	Motor vehicle collision with C5/6 spinal fracture
CZEEOQ	Bilateral transfemoral amputations, left index finger amputation, colostomy, tympanic membrane damage, ureter reconstruction , pelvic fracture repair, perineum repair, urethral reconstruction
D9NU7Z	
EGA3QF	Left pleural effusion, right pulmonary embolus, status post laparotomy with hepatectomy for severe liver injury with bile leak, multiple bilateral rib fractures, delirium, burn wounds
EQ4PON	
FYR1CA	66% scalding burn to face, torso, bilateral upper extremities, bilateral lower extremities and genitals
H02X39	Fall from standing with spontaneous bleed
HPBY51	
M7OWC1	Motor vehicle accident, right upper traumatic amputation
MSA70S	Pelvic Fracture, perineal laceration, retroperitoneal hematoma, scrotal evisceration with left orchiectomy, nosocomial pneumonia
SK76NU	9% TBSA flame burns from oil rig explosion; burn to right and Left arms/legs, face, back, buttocks
UFIXCO	Motor vehicle collision, head on collision with 18 wheeler
ZCNKD5	Small bowel obstruction

Table S2: Piperacillin Model Building

Run	Description	Algorithm	OFV	#Param	Decision
**Base Model**					
1	1cmpt prop error	FOCEI	594.22	5	-
2	2cmpt prop error	FOCEI	559.03	7	Accept
3	2cmpt prop + add error	FOCEI	559.03	8	Reject
**Covariate Model Using 2 as Base**					
**Clearance Covariates**					
4	As 2 + isBURN on CL	FOCEI	558.12	8	Reject
5	As 2 + TBSA on CL	FOCEI	558.13	8	Reject
6	As 2 + WTSAM on CL	FOCEI	554.68	8	Accept[1]
7	As 2 + LBM on CL	FOCEI	554.72	8	Reject
8	As 2 + CRCL on CL	FOCEI	552.3	8	Accept[2]
9	As 2 + UOP on CL	FOCEI	553.83	8	Accept[3]
10	As 8 + UOP on CL	FOCEI	548.94	9	Reject[4]
1. P = 0.037, exponent estimate = 0.81, explains 5.91% variability on CL (%CV)<br>2. P = 0.009, exponent estimate = 0.65, explains 8.71% variability on CL (%CV)<br>3. P = 0.023, linear model, explains 6.98% variability on CL (%CV)<br>4.Trend of P = 0.067					
**Volume Covariates**					
11	As 2 + isBURN on Vc	FOCEI	558.14	8	Reject
12	As 2 + isBURN on Vp	FOCEI	557.74	8	Reject
13	As 2 + TBSA on Vc	FOCEI	556.58	8	Reject
14	As 2 + TBSA on Vp	FOCEI	557.18	8	Reject
15	As 2 + WTSAM on Vc	FOCEI	558.71	8	Reject
16	As 2 + LBM on Vc	FOCEI	558.19	8	Reject
17	As 2 + ALBUM on Vc	FOCEI	558.95	8	Reject
18	As 2 + ALBUM on Vp	FOCEI	555.93	8	Reject[1]
1. Trend towards significance p = 0.078, exponent estimate -1.61, BSV on Vp not estimated.					

Table S3: Tazobactam Model Building

Run	Description	Algorithm	OFV	#Param	Decision
**Base Model**					
1	1cmpt prop error	FOCEI	290	5	-
2	2cmpt prop error	FOCEI	266.3	7	Accept
3	2cmpt prop + add error	FOCEI	266.3	8	Reject
**Covariate Model Using 2 as Base**					
**Clearance Covariates**					
4	As 2 + isBURN on CL	FOCEI	265.63	8	Reject
5	As 2 + TBSA on CL	FOCEI	266.28	8	Reject
6	As 2 + WTSAM on CL	FOCEI	264.44	8	Reject
7	As 2 + LBM on CL	FOCEI	265.04	8	Reject
8	As 2 + CRCL on CL	FOCEI	254.96	8	Accept[1]
9	As 9 without eta Vc	FOCEI	255.22	6	Accept[2,3]
10	As 2 + UOP on CL	FOCEI	264.14	6	Reject
1. P = 0.001, exponent estimate 0.77, explains 23.68% variability on CL (%CV).<br>2. Eta shrinkage on Vc 99.9% in run 8, therefore removed estimate of etaVc from model.<br>3. Given previous estimate of 0.77 similar to 0.67 from Chandorkar et al, fixed CrCl estimate to 0.67.					
**Volume Covariates**					
10	As 2 + isBURN on Vc	FOCEI	265.38	8	Reject
11	As 2 + isBURN on Vp	FOCEI	265.97	8	Reject
12	As 2 + TBSA on Vc	FOCEI	265.58	8	Reject
13	As 2 + TBSA on Vp	FOCEI	265.98	8	Reject
14	As 2 + WTSAM on Vc	FOCEI	266.3	8	Reject
15	As 2 + LBM on Vc	FOCEI	266.3	8	Reject
16	As 2 + ALBUM on Vc	FOCEI	266.3	8	Reject
17	As 2 + ALBUM on Vp	FOCEI	263.41	8	Reject[1]
1. Trend of P = 0.089, estimated effect size exponent = -3.34.					

Table S4: Sensitivity Analysis of Fraction Unbound on Probability of Target Attainment

**PTA Target 50% fT > MIC 16 mg/L**				
**Fraction Unbound**	**Continuous Infusion Dose**			
	12 g/day	16 g/day	24 g/day	28 g/day
0.7	99%	100%	100%	100%
0.6	98%	100%	100%	100%
0.5	95%	99%	100%	100%
**PTA Target 100% fT > MIC 16 mg/L**				
**Fraction Unbound**	**Continuous Infusion Dose**			
	12 g/day	16 g/day	24 g/day	28 g/day
0.7	99%	100%	100%	100%
0.6	98%	100%	100%	100%
0.5	95%	99%	100%	100%
**PTA Target 100% fT > 4×MIC 16 mg/L**				
**Fraction Unbound**	**Continuous Infusion Dose**			
	12 g/day	16 g/day	24 g/day	28 g/day
0.7	18%	39%	76%	87%
0.6	8.3%	27%	63%	77%
0.5	2.9%	16%	46%	62%

Figure S1: Clearance Covariate Explorations for Piperacillin (left) and Tazobactam (right). Bottom row only shows trends from base models.

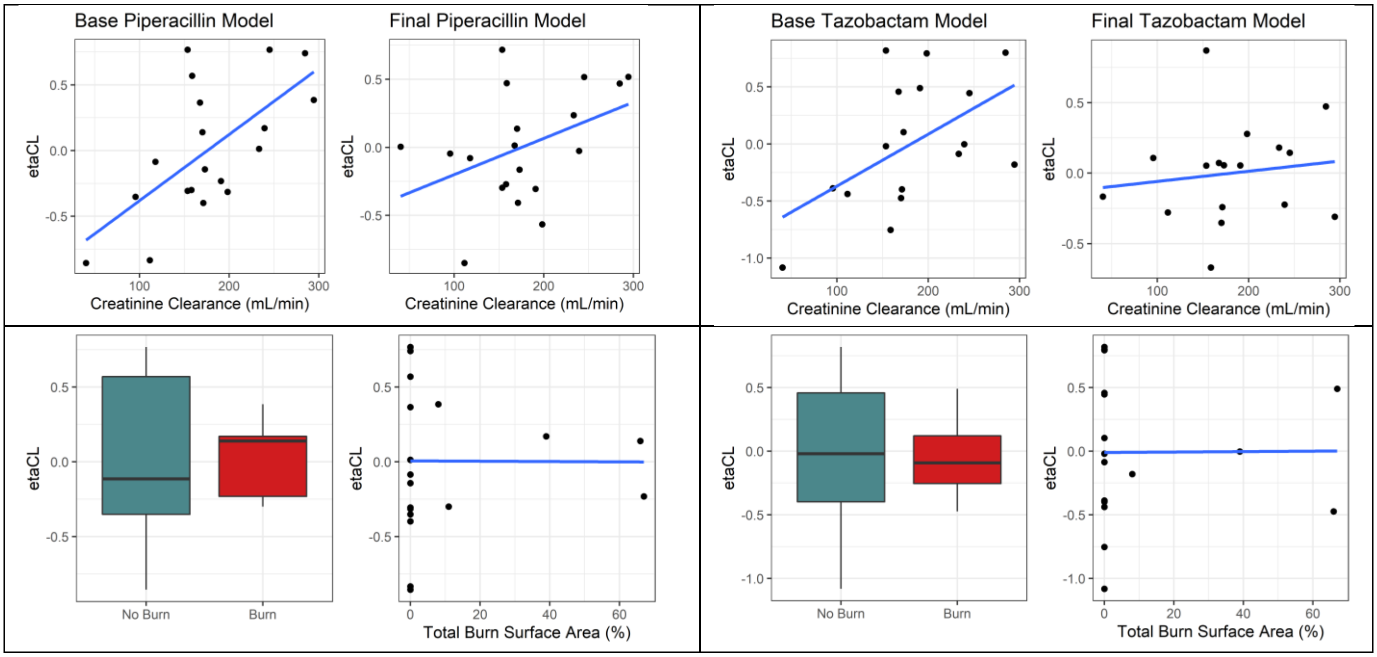

Figure S2: Volume Covariate Explorations for Piperacillin (left) and Tazobactam (right) (base models)

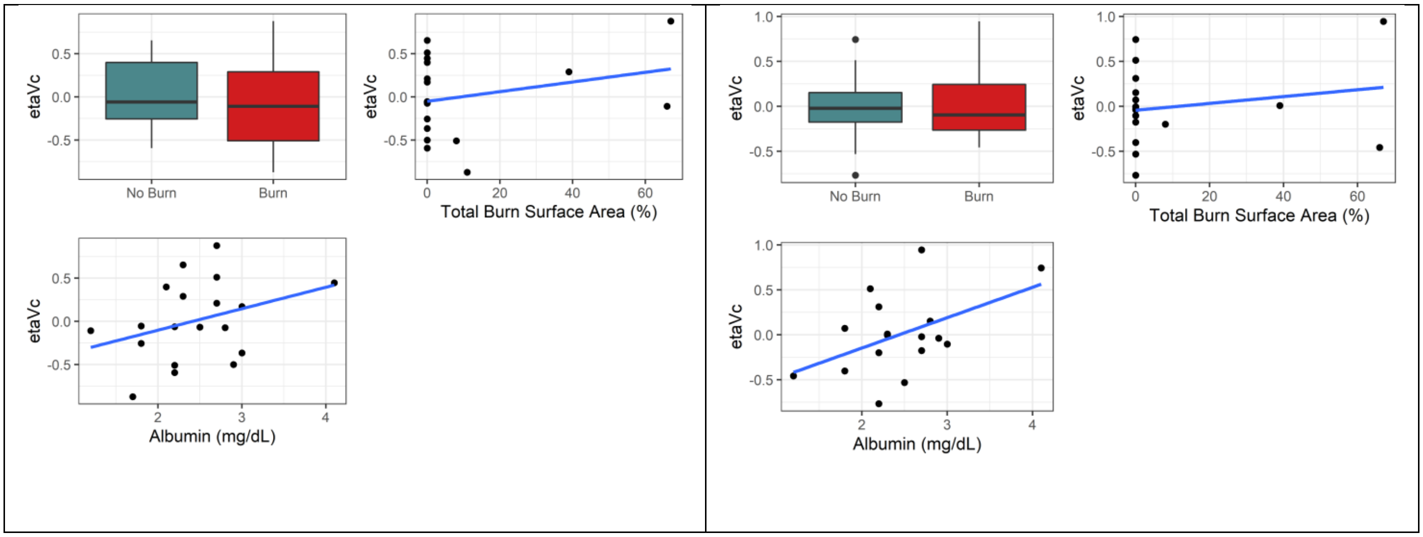

Figure S3: Individual Goodness of Fit Plots (top piperacillin, bottom tazobactam)

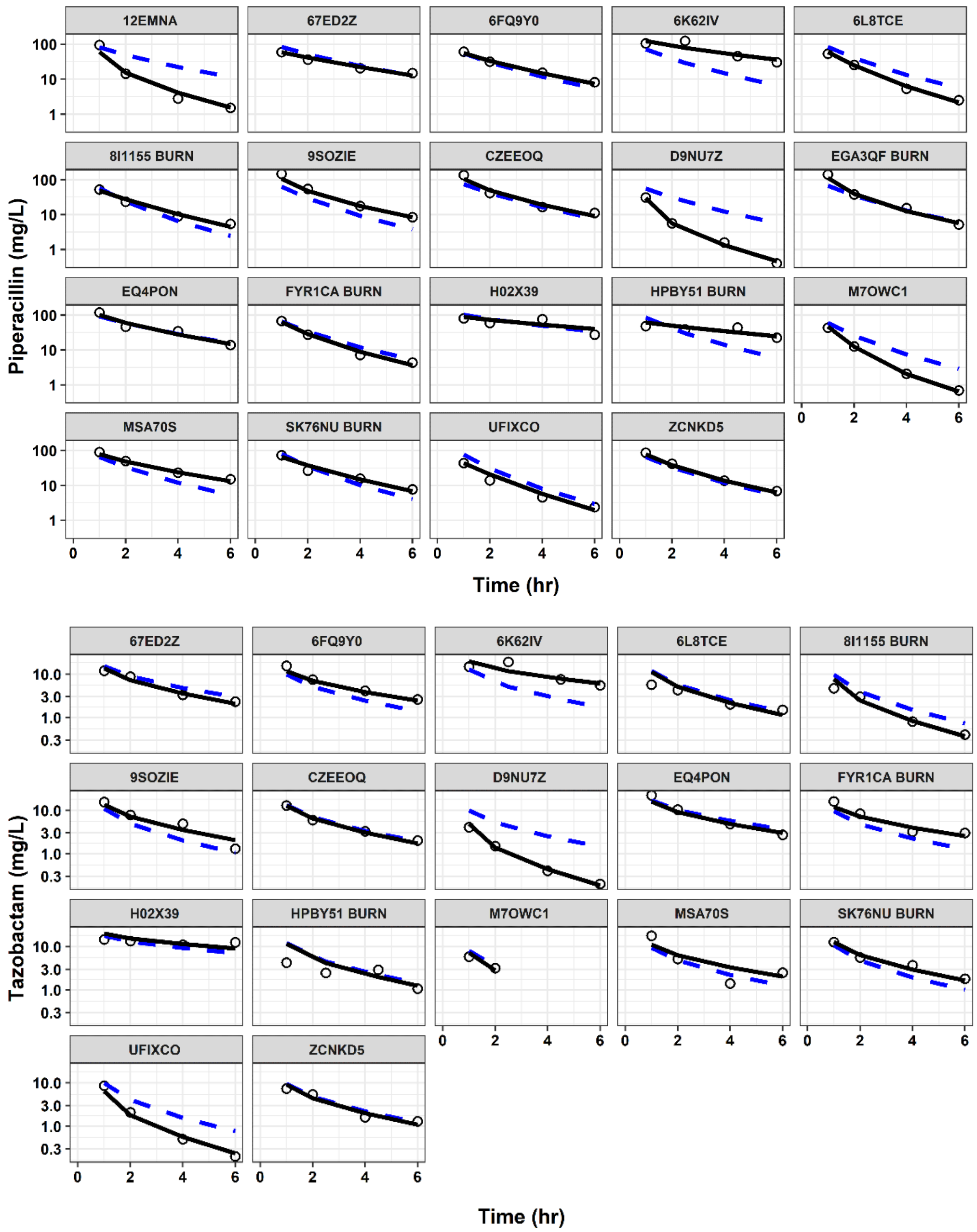

Blue dashed lines = population prediction, black solid lines = individual predictions

Figure S4: Summary of Residual and Between Subject Variabilities (top row piperacillin, bottom row tazobactam)

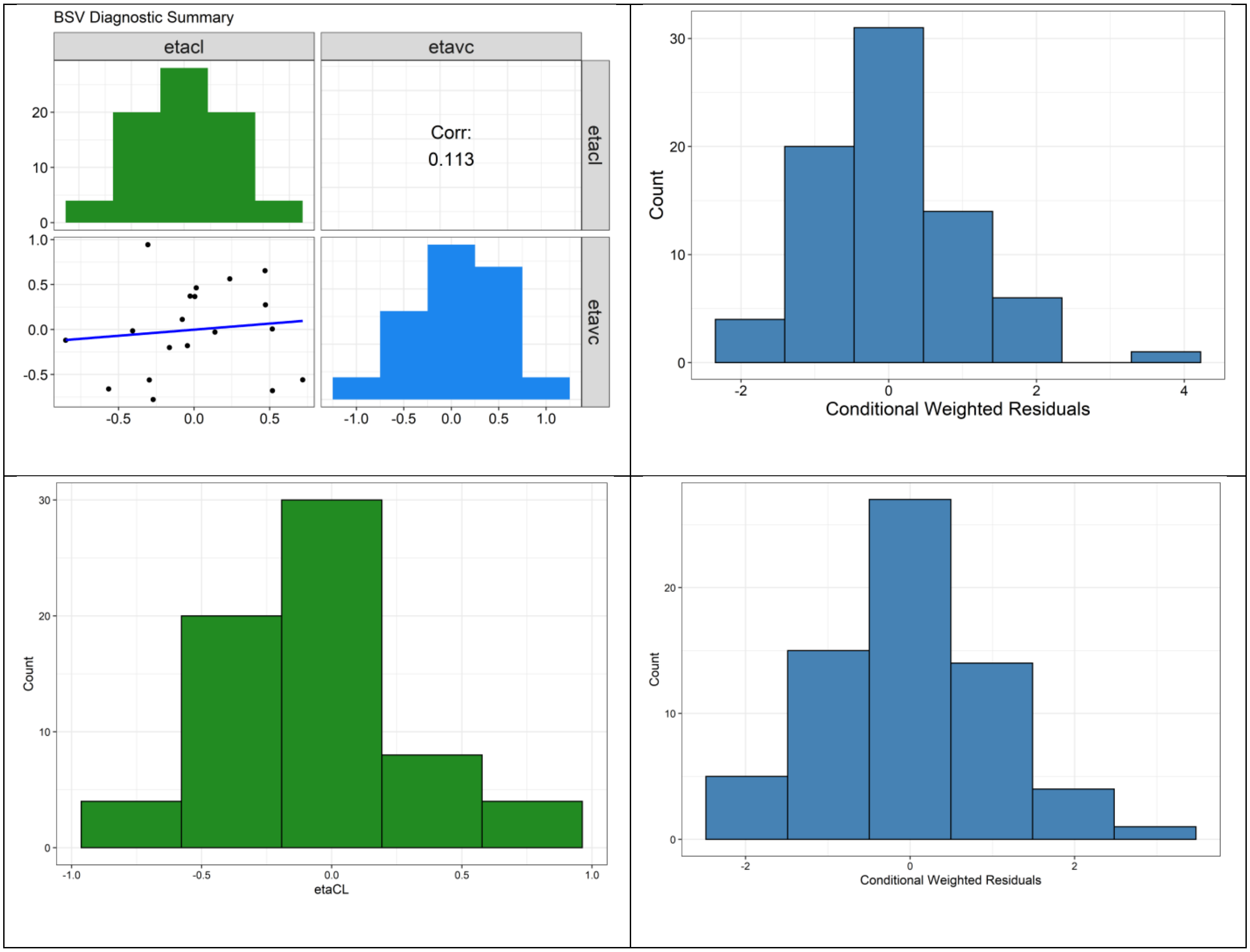

Figure S5: Visual Predictive Checks for Piperacillin (left) and Tazobactam (right). Blue circles represent observed data, red lines represent observed data quantiles (0.1, 0.5, 0.9), and black lines represent simulated data quantiles (0.1, 0.5, 0.9).

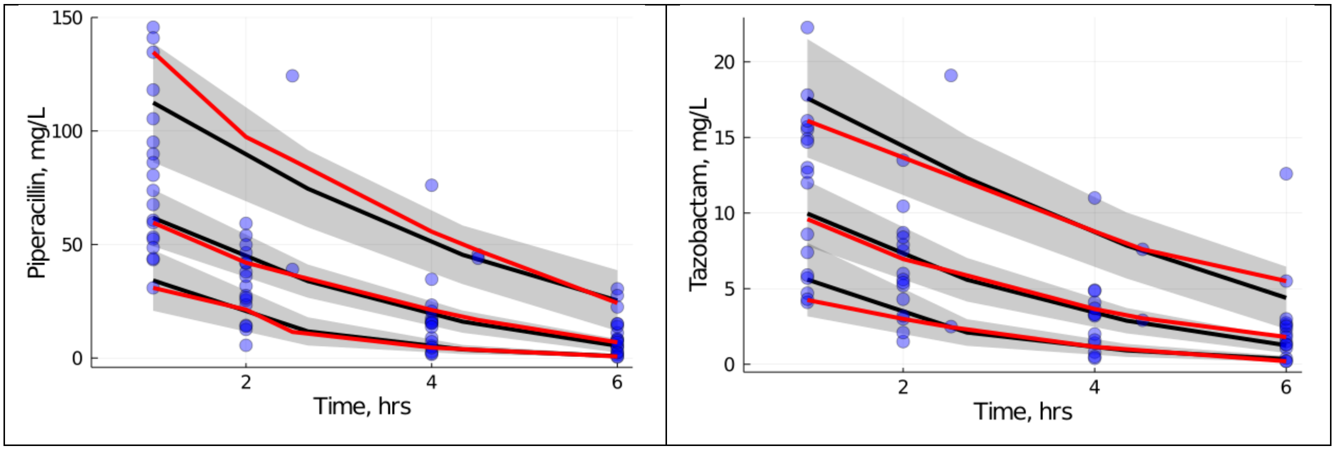

Supplement: Supplementary file 1 [file antibiotics-11-00618-s001.zip › antibiotics-1682753-supplementary.pdf]
